# Supplementary figures and images for: Simple Sequence Repeat Markers Reveal Genetic Diversity and Population Structure of Bolivian Wild and Cultivated Tomatoes (Solanum lycopersicum L.)
Source: Genes (Basel). 2022 Aug 23;13(9):1505. doi: 10.3390/genes13091505 (PMC9498693; doi:10.3390/genes13091505)

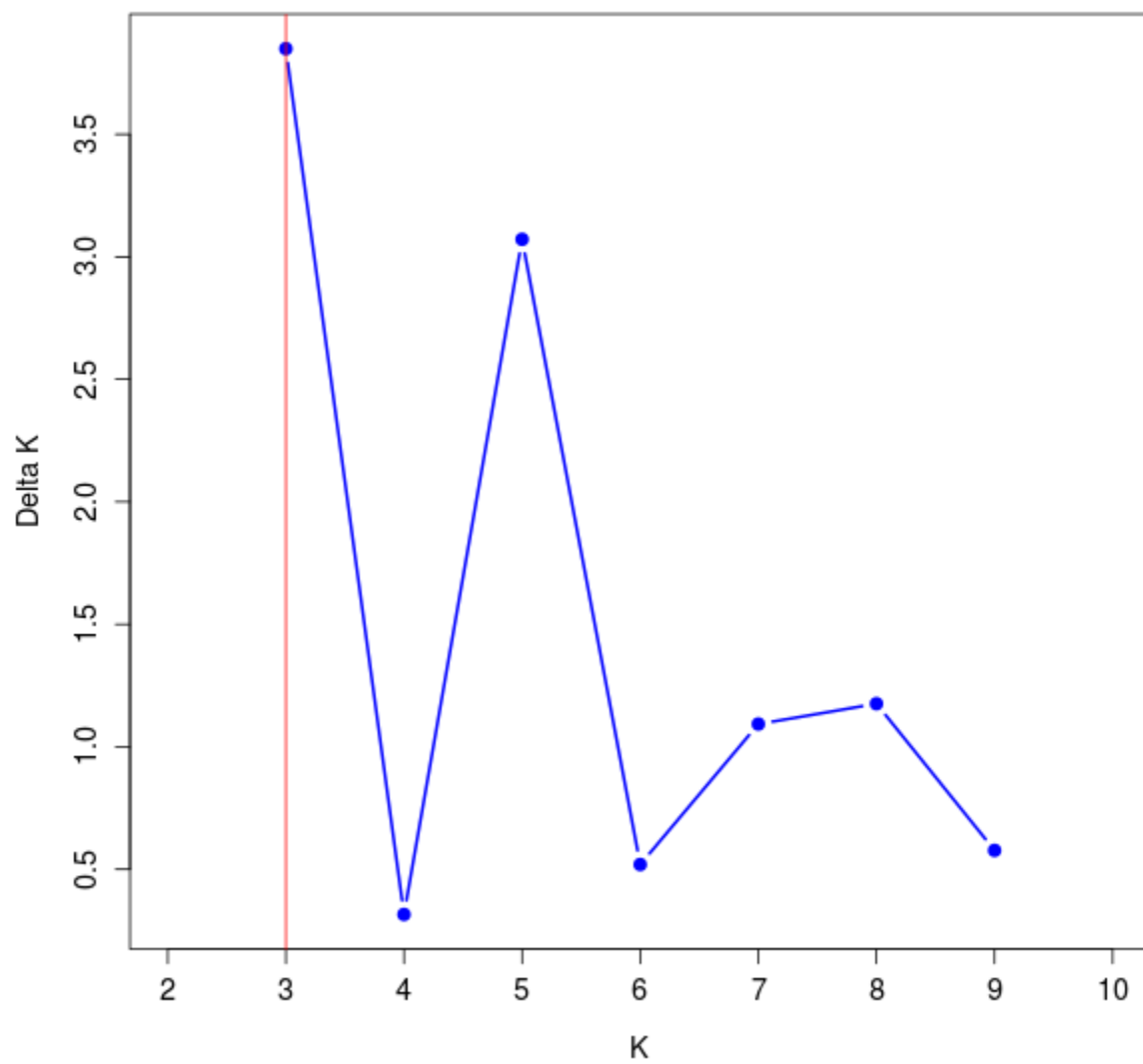

**Figure S1.** Delta-K plot with a maximum value at 3 ( $\Delta K=3$ ).

Supplement: Supplementary file 1 [file genes-13-01505-s001.zip › Supp fig and table 2022 08 11/Suplementary Figure S1.pdf]
